# Determination of Plasma Potential Using an Emissive Probe with Floating Potential Method

**DOI:** 10.3390/ma16072762

**Published:** 2023-03-30

**Authors:** Chulhee Cho, Sijun Kim, Youngseok Lee, Inho Seong, Wonnyoung Jeong, Yebin You, Minsu Choi, Shinjae You

**Affiliations:** 1 Applied Physics Lab for PLasma Engineering (APPLE), Department of Physics, Chungnam National University, Daejeon 34134, Republic of Korea; 2 Institute of Quantum Systems (IQS), Chungnam National University, Daejeon 34134, Republic of Korea

**Keywords:** plasma diagnostics, plasma potential, emissive probe, floating potential method, plasma potential determination

## Abstract

Despite over 90 years of study on the emissive probe, a plasma diagnostic tool used to measure plasma potential, its underlying physics has yet to be fully understood. In this study, we investigated the voltages along the hot filament wire and emitting thermal electrons and proved which voltage reflects the plasma potential. Using a circuit model incorporating the floating condition, we found that the lowest potential on the plasma-exposed filament provides a close approximation of the plasma potential. This theoretical result was verified with a comparison of emissive probe measurements and Langmuir probe measurements in inductively coupled plasma. This work provides a significant contribution to the accurate measurement of plasma potential using the emissive probe with the floating potential method.

## 1. Introduction

Plasma, composed of charged and neutral particles, has been widely used in material processing, since it provides physically energetic ion bombardment and chemically reactive species on the material surface. In particular, plasma has played a significant role in plasma processes such as plasma etching [1,2,3], ashing [4], deposition [5], and plasma decomposition [6,7]. To analyze the process chemistry and mechanism, several instruments have been developed and utilized, such as voltage–current probes [8,9], optical emission spectroscopy [10,11], and quadrupole mass spectrometers [12,13]. Recently, understanding plasma behavior in plasma processing has attracted significant attention, since plasma produces chemical species and thus dominates process chemistry [14].

Basic internal plasma parameters are related with charged particles, called electron density, ion energy, and plasma potential. Various diagnostic tools have been developed to measure internal plasma parameters, such as microwave probes for electron density [15,16], ion energy analyzers [17,18] for ion energy, and electrostatic probes [19,20] for plasma potential. Among these parameters, plasma potential is a crucial parameter, since it confines electrons and dominates the flow dynamics of ions in plasma [21].

The emissive probe, which has been studied for over 90 years, is a precise diagnostic tool for measuring plasma potential [20]. It has a hot filament emitting thermal electrons into plasma, which decreases the potential difference between plasma and filament; then, measuring the filament voltage provides an estimation of plasma potential. There are three types of methods for the determination of plasma potential with the emissive probe [20]: (i) differential, (ii) inflection-point, and (iii) floating potential methods. The differential method employs two emissive probes, called cold and hot probes [22]. By sweeping the voltage of two probes and measuring their current, the differential method determines the plasma potential as the voltage at the separation point where their currents cross. The inflection-point method adopts one emissive probe and determines the plasma potential as the peak in the first derivatives of the measured current–voltage curve [23]. The floating potential method uses one emissive probe that is electrically floated with plasma [24,25]. With strong electron emission equal to the incoming electron flux from plasma, its floating potential approximates to the plasma potential; thus, this method determines the plasma potential as the floating potential.

Compared with other methods, the floating potential method is regarded to be more effective and convenient for measuring plasma potential due to its relatively simple system elements and capability for time-transient measurements [25,26,27,28,29,30]. Despite considering its long history, its underlying physics has yet to be fully understood. In 1966, Kemp et al. [24] introduced the floating potential method with an emissive probe and proved that the floating potential approaches the plasma potential with the increase in filament temperature, which leads to greater emission from the filament. After that, most studies have focused on the space charge effect on the emissive probe and plasma [20,31,32]. A strong emission flux larger than the electron flux from plasma forms extra space charges, and a virtual cathode forms in front of the emitting surface [20], which deteriorates the accuracy of the emissive probe measurement. In addition to the space charge limit condition, another factor that affects the accuracy has been recently reported. Jílek et al. reported a computational study of the voltage distribution on the filament surface along with the filament temperature distribution [32]. They found that the floating voltage on the filament differs from the plasma potential due to the voltage distribution induced by temperature, which might decrease the measurement accuracy. Besides the temperature distribution effect, the voltage drop due to filament resistance along a filament wire also enables the voltage distribution to be formed, but its effect has yet to be investigated. Hence, in this study, we investigated voltages along the emitting filament surface with respect to voltage drop due to filament resistance and proved which potential reflects the plasma potential using a circuit model and an experimental demonstration.

This paper is structured as follows: Section 2 provides a comprehensive description of the circuit model of the emissive probe and presents an analysis of the voltage characteristics of the emissive probe. Section 3 describes the experimental setup and discusses the results of emissive probe and Langmuir probe measurements. Finally, the concluding section presents a summary of the findings presented in this paper.

## 2. Circuit Model Analysis

### 2.1. Circuit Model Details

**Figure 1:**
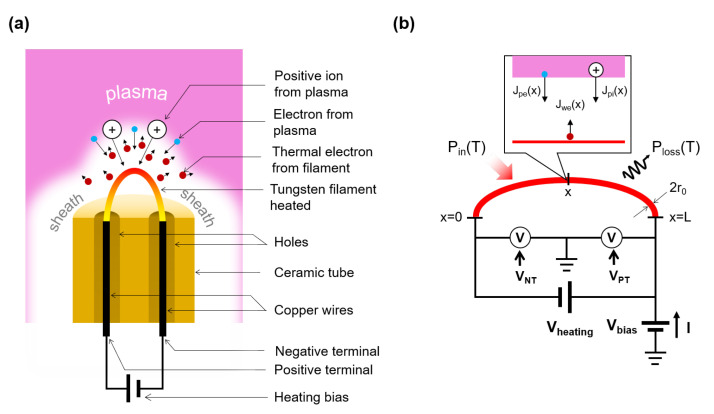
Schematic diagrams of (**a**) an emissive probe system using floating potential method and (**b**) a circuit model of the emissive probe.

In this section, we briefly explain the configuration of the emissive probe and then describe the circuit model in detail. Figure 1 shows the schematic diagram of an emissive probe composed of a ceramic tube, a tungsten filament, and copper wires. The ceramic tube has two holes that isolate the copper wires from each other. The heating bias connected with the copper wires allows current to flow, which makes it possible to heat the tungsten filament, which emits thermal electrons. Here, the power dissipated in the filament dominates the heating bias power, as the copper wire has lower resistance than the tungsten filament. In addition to the emitted electrons entering plasma, electrons and positive ions from plasma also come into the filament. Since with the floating potential method, the emissive probe is electrically floating, the total current formed by charged particles (emitted electrons, electrons, and positive ions from plasma) is zero.

Figure 1b shows a schematic diagram of the circuit model. The tungsten filament is connected with a heating source (V${}_{\mathrm{heating}}$), and this system has a bias potential (V${}_{\mathrm{bias}}$) satisfying the floating condition. With constant V${}_{\mathrm{heating}}$, the current (*I*) flowing through the filament is determined as

(1) $$I=\frac{V_{\mathrm{heating}}}{R_{W}\left(T\left(x\right)\right)},$$

where R${}_{W}$(T(*x*)) is the tungsten filament resistance depending on the filament temperature (T(*x*)) at position *x*, defined as

(2) $$R\left(T(x)\right)=\frac{\rho_{W}\left(T\left(x\right)\right)\times L}{A},$$

where $\rho_{W}$(T(*x*)) is the temperature-dependent filament resistivity [33], L is the filament length, A (=2$\pi r_{0}\times L$) is the filament area, and $r_{0}$ is the filament radius. Here, the copper wire resistance is neglected, since it is lower than that of the filament. The voltage drop along the filament ($\Delta V_{W}\left(x\right)$) within Δx is defined as

(3) $$\Delta V_{W}\left(x\right)\equiv V_{W}\left(x+\Delta x\right)-V_{W}\left(x\right)=I\times \Delta R\left(T\left(x\right)\right)=I\times \frac{\rho_{W}\left(T\left(x\right)\right)\times \Delta x}{A}.$$

The filament temperature is determined with the power balance equation as

(4) $$P_{\mathrm{in}}\left(T\left(x\right)\right)=P_{\mathrm{loss}}\left(T\left(x\right)\right),$$

where P${}_{\mathrm{in}}$(T(*x*)) is the input power of ohmic heating and P${}_{\mathrm{loss}}$(T(*x*)) is the lost power, including Stefan–Boltzmann radiation and thermal conduction loss. The input power and lost power are defined as

(5) $$P_{\mathrm{in}}\left(T\left(x\right)\right)=I^{2}\times R\left(T\left(x\right)\right),$$

(6) $$P_{\mathrm{loss}}\left(T\left(x\right)\right)=\sigma_{W}T^{4}\left(x\right)+n_{W}c_{W}\frac{\mathrm{dT}(x)}{dx},$$

respectively, where $\sigma_{W}$ is the emissivity of a tungsten [33], $n_{W}$ is the tungsten mass density, and $c_{W}$ is the heat capacity under constant pressure. Here, we assumed uniform filament temperature, that is, T(*x*) = T, for the clear analysis of the voltage drop effect induced by filament resistance; thus, the thermal conduction loss is neglected when calculating P${}_{\mathrm{loss}}$, that is, dT/d*x* = 0.

After the filament temperature is settled, V${}_{\mathrm{bias}}$ is determined using the macroscopic floating condition of the filament. Since the floating condition implies zero total charged particle current, those current densities on the whole filament wire become balanced as

(7) $$\frac{1}{L}\int_{0}^{L}J_{\mathrm{pe}}\left(x\right)dx=\frac{1}{L}\int_{0}^{L}J_{\mathrm{we}}(T\left(x\right)=T)dx+\frac{1}{L}\int_{0}^{L}J_{\mathrm{pi}}\left(x\right)dx,$$

where $J_{\mathrm{pe}}$(*x*) is the electron current density from plasma, $J_{\mathrm{we}}$(T(*x*) = T) is the emitted electron current density, and $J_{\mathrm{pi}}\left(x\right)$ is the ion current density from plasma, as depicted in Figure 1. Those current densities depend on the relation between the voltage on the filament ($V_{W}\left(x\right)$) and plasma potential ($V_{p}\left(x\right)$). At $V_{W}\left(x\right)<V_{p}\left(x\right)$, $J_{\mathrm{pe}}$ is defined by the Boltzmann relation [14] as

(8) $$J_{\mathrm{pe}}\left(x\right)=\frac{1}{4}ev_{\mathrm{th}}\left(T_{e}\left(x\right)\right)n_{e}\left(x\right)\exp\left(\frac{e\left(V_{W}\left(x\right)-V_{p}\left(x\right)\right)}{T_{e}\left(x\right)}\right),$$

where *e* is the elementary charge, $v_{\mathrm{th}}\left(=\sqrt{3k_{B}T_{e}\left(x\right)/m_{e}}\right)$ is the thermal velocity of electrons, $k_{B}$ is the Boltzmann constant, $m_{e}$ is the electron mass, $T_{e}\left(x\right)$ is the electron temperature, and $n_{e}\left(x\right)$ is the electron density. At $V_{W}\left(x\right)>V_{p}\left(x\right)$, Equation (8) becomes

(9) $$J_{\mathrm{pe}}\left(x\right)=\frac{1}{4}ev_{\mathrm{th}}\left(T_{e}\left(x\right)\right)n_{e}\left(x\right),$$

since all plasma electrons are attracted to the filament surface. $J_{\mathrm{we}}$(T) also depends on the relation between $V_{W}\left(x\right)$ and $V_{p}\left(x\right)$. At $V_{W}\left(x\right)<V_{p}\left(x\right)$, it is calculated with the Richardson–Dushman equation, defined as

(10) $$J_{\mathrm{we}}\left(T\right)=\left(\frac{4\pi em_{e}k_{B}^{2}}{h^{3}}\right)T^{2}\exp\left(-\frac{\Phi_{W}}{k_{B}T}\right),$$

where *h* is the Planck constant and $\Phi_{W}$ is the work function of tungsten ($\Phi_{W}$ = 4.54 eV) [34]. In this regime, all thermionic electrons are attracted to plasma. At $V_{W}\left(x\right)>V_{p}\left(x\right)$, Equation (10) becomes

(11) $$J_{\mathrm{we}}\left(T\right)=\left(\frac{4\pi em_{e}k_{B}^{2}}{h^{3}}\right)T^{2}\exp\left(-\frac{\Phi_{W}}{k_{B}T}\right)\exp\left(\frac{e\left(V_{W}\left(x\right)-V_{p}\left(x\right)\right)}{T_{\mathrm{we}}}\right),$$

where $T_{\mathrm{we}}$ is the temperature of the emitted electrons. Regarding J${}_{\mathrm{pi}}\left(x\right)$, it is defined with the Bohm flux [14], as at $V_{W}\left(x\right)<V_{p}\left(x\right)$,

(12) $$J_{\mathrm{pi}}\left(x\right)=n_{i}\left(x\right)u_{B}\left(x\right),$$

where $n_{i}\left(x\right)$ is the ion density, $u_{B}\left(x\right)\left(=\sqrt{eT_{e(x)}/m_{i}}\right)$ is the Bohm velocity, and $m_{i}$ is the mass of the ion. At $V_{W}\left(x\right)>V_{p}\left(x\right)$, Equation (12) becomes

(13) $$J_{\mathrm{pi}}\left(x\right)=0,$$

since the ion kinetic energy inside plasma is about 0.026 eV [14], which corresponds to the room temperature, and it is too small to overcome the potential barrier, $e(V_{W}\left(x\right)-V_{p}\left(x\right))$, which ranges a few volts.

Furthermore, the plasma parameters can be assumed as homogeneous along the filament wire, that is, $T_{e}\left(x\right)=T_{e}$, $n_{e}\left(x\right)=n_{e}$, and $V_{p}\left(x\right)=V_{p}$, for simplicity.

### 2.2. Results and Discussion

**Figure 2:**
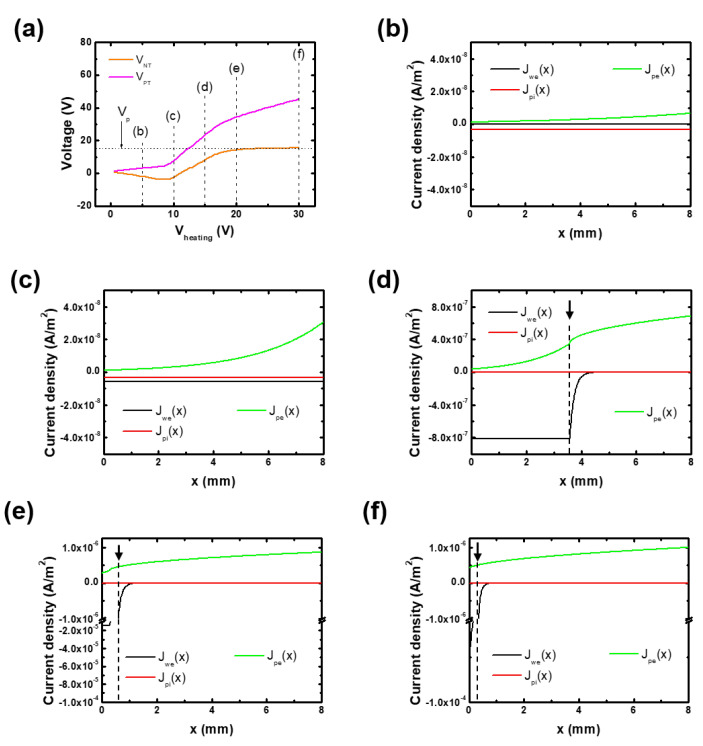
(**a**) Calculated negative and positive terminal voltages (V${}_{\mathrm{NT}}$ and V${}_{\mathrm{PT}}$) in the circuit model. The dashed line indicates the plasma potential. (**b**–**f**) Calculated charged particle current densities over the filament wire at different heating voltages: (**b**) 5 V, (**c**) 10 V, (**d**) 15 V, (**e**) 20 V, and (**f**) 30 V. The simulation parameters were as follows: plasma potential of 15 V, electron density of 1.0 × 10${}^{10}$ cm${}^{-3}$, electron temperature of 3.0 eV, filament length of 8.0 mm, filament diameter of 15 µm, and thermionic electron temperature (T${}_{w}$) of 0.3 eV.

For the validation of our circuit model, we calculated the negative terminal voltage (V${}_{\mathrm{NT}}$) at x=0 and the positive terminal one (V${}_{\mathrm{PT}}$) at x=L depicted in Figure 1 and compared them with previous results. Here, these voltages are the common parameters of emissive probes with the floating potential method. Figure 2a shows the calculated V${}_{\mathrm{PT}}$ and V${}_{\mathrm{NT}}$ against V${}_{\mathrm{heating}}$. V${}_{\mathrm{PT}}$ gradually increases against V${}_{\mathrm{heating}}$, whereas V${}_{\mathrm{NT}}$ is saturated to the plasma potential. In [24,32], the floating potential approaches the plasma potential with the increase in the heating voltage (or filament temperature). This saturation trend against V${}_{\mathrm{heating}}$ is well reproduced in the circuit model, as shown in Figure 2a.

The circuit model result reveal that the negative terminal voltage is close to the plasma potential rather than the positive one. To understand this, we analyzed the charged particle current densities along the filament wire at various V${}_{\mathrm{heating}}$, and they are represented in Figure 2b–f.

At low heating voltage (V${}_{\mathrm{heating}}<10$ V), the small current flows through the filament (Equation (1)), and it induces low filament temperatures and near-zero J${}_{\mathrm{we}}$, as shown in Figure 2b,c. In this regime, $V_{\mathrm{PT}}$ and $V_{\mathrm{NT}}$ gradually increase and decrease, respectively, with the increase in $V_{\mathrm{heating}}$.

At sufficient heating voltage (V${}_{\mathrm{heating}}>10$ V), making it possible to emit thermal electrons, $V_{\mathrm{PT}}$ steeply rises beyond V${}_{p}$, while $V_{\mathrm{NT}}$ increases and approaches V${}_{p}$. The increase in the two terminal voltages results from the increase in V${}_{\mathrm{bias}}$. As J${}_{\mathrm{we}}$ is effective under the floating condition, as in Equation (7), at high V${}_{\mathrm{bias}}$, the floating potential increases to balance the floating condition; thus, V${}_{\mathrm{bias}}$ increases. In this regime, J${}_{\mathrm{we}}\left(x\right)$ is released within the critical distance, marked by the arrow and the dashed line, and it becomes zero above the critical distance. This means that V${}_{W}\left(x\right)$ becomes larger than V${}_{p}$ above the critical distance, which results in the transition of J${}_{\mathrm{we}}\left(x\right)$ from Equation (10) to Equation (11); since the voltage difference, V${}_{W}\left(x\right)-$ V${}_{p}$, is much larger than T${}_{\mathrm{we}}$, J${}_{\mathrm{we}}$ approaches the zero, as shown in Figure 2d. As the heating voltage increases, the critical distance approaches the negative terminal position (x=0), as shown in Figure 2e,f. Hence, V${}_{\mathrm{NT}}$ approaches the plasma potential, as shown in Figure 2a.

Moreover, at high V${}_{\mathrm{heating}}$, only a small region below the critical distance emits thermal electrons with high intensity due to high current (I), and the floating condition is saturated, which results in the saturation of V${}_{\mathrm{NT}}$ to V${}_{p}$. Under this condition, V${}_{\mathrm{PT}}$ linearly increases with the increase in V${}_{\mathrm{heating}}$ due to the fixed V${}_{\mathrm{NT}}$.

**Figure 3:**
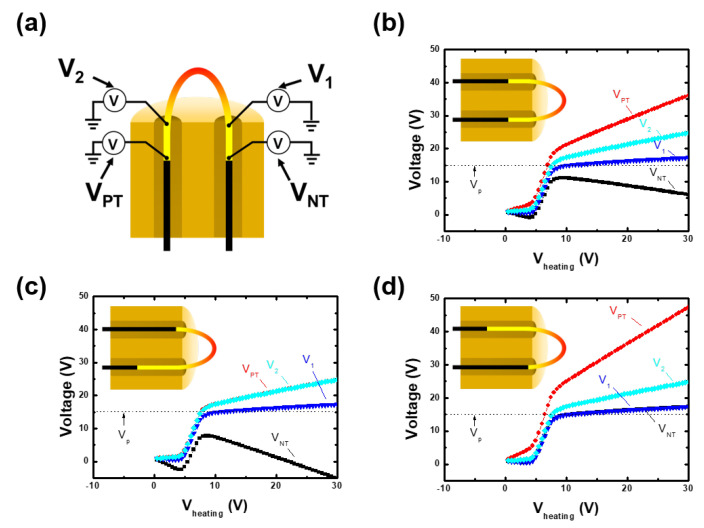
(**a**) Schematic diagram of emissive probe and voltage measurement (V${}_{1}$, V${}_{2}$, V${}_{\mathrm{PT}}$, and V${}_{\mathrm{NT}}$) positions in the circuit model. Calculated voltages against heating voltage (V${}_{\mathrm{heating}}$) with (**b**) symmetric, (**c**) right-sided, and (**d**) left-sided filament configurations. The simulation parameters were as follows: plasma potential of 15 V, electron density of 1.0 × 10${}^{10}$ cm${}^{-3}$, electron temperature of 3.0 eV, total filament length of 20.0 mm, exposed filament length of 5.0 mm, filament diameter of 0.25 mm, and thermionic electron temperature (T${}_{w}$) of 0.3 eV.

However, the whole filament wire is not exposed to plasma in practical use, since some filament regions are inserted in the ceramic tube. Figure 3a shows a schematic diagram of realistic filament configurations used in the circuit model. We investigated three types of filament configurations, symmetric, and right- and left-sided filament configurations, which are commonly used in practical use. To simulate the shielding effect by the ceramic tube in the circuit model, the voltage dropped in the filament (Equation (3)) included this region, but the floating condition (Equation (7)) excluded it, since thermionic emission due to charge accumulation is not permitted in the ceramic tube in this region. Furthermore, we calculated the voltages at the filament edges (V${}_{\mathrm{PT}}$ and V${}_{\mathrm{NT}}$) and at the edges of the exposed region (V${}_{1}$ and V${}_{2}$).

Figure 3b–d show filament configurations and voltage characteristics in three cases. The results exhibit that V${}_{1}$ approaches V${}_{p}$. Considering that the positive terminal-sided voltage is always higher than the negative-sided one, we can conclude that the lowest voltage along the plasma-exposed filament region is close to the plasma potential at sufficient heating voltage.

Furthermore, this result implies that the filament configuration is also a key factor in measurement accuracy. Shortening the length where the filament is shielded by the ceramic tube lowers the measurement discrepancy.

## 3. Experimental Validation

### 3.1. Experimental Setup

**Figure 4:**
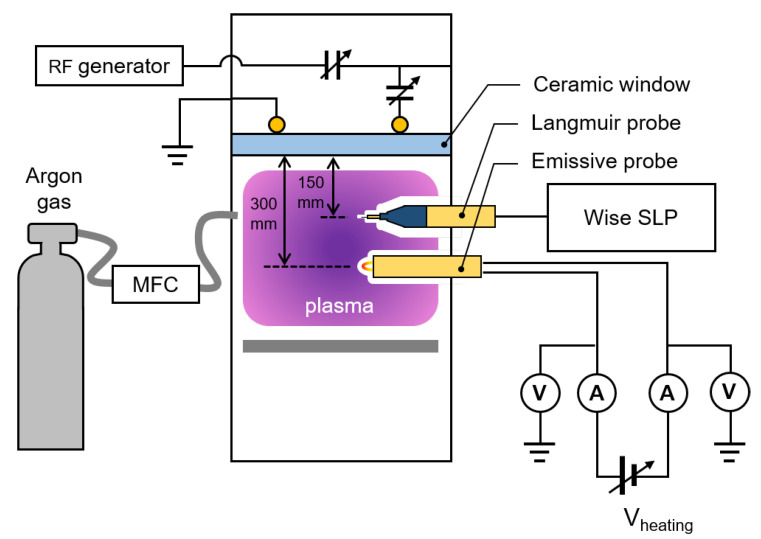
Schematic diagram of experimental setup for inductively coupled plasma system with an emissive probe and a Langmuir probe.

To validate the simulation results, we compared the emissive probe measurement with Langmuir probe measurement in an inductively coupled plasma (ICP) source. Figure 4 shows a schematic diagram of the experimental setup. For plasma generation, the 13.56 MHz radio-frequency (RF) power of 200 W from an RF generator (YSR-06MF; YongSin RF Inc., Hanam-si, Korea) was applied to an inductive coupling one-turn antenna using an RF matcher (YongSin RF Matcher; YongSin RF Inc., Hanam-si, Korea). Argon gas (99.999% purity) at 10 standard cubic centimeter per minutes (sccm) was injected using a mass flow controller (MFC; LineTech Inc., Deajeon, Korea). A rotary pump (DS102; Agilent Inc., Santa Clara, CA, USA) drew Argon gas to sustain the chamber pressure of 10 mTorr.

The emissive probe was inserted into the ICP chamber at the center at a distance of 300 mm from the ceramic antenna. A tungsten filament with a diameter of 0.25 mm and a total length of 30 mm was used. In this case, the exposed filament length was 5.0 mm. The DC power supply (KSC-G; Korea Switching, Seongdong-gu, Seoul, Korea) was used to output heating voltage and current. The DC power supply was electrically floated with the ground in this system. We measured the terminal voltages and currents using digital multi-meters (15B+ DIGITAL MULTIMETER; FLUKE Co., Everett, WA, USA).

The Langmuir probe was inserted into the chamber at the center at a distance of 150 mm from the ceramic window. We used in-house RF chokes for the RF compensation of the first harmonics from plasma potential oscillation [35]. The tungsten wire tip had a length of 2.0 mm and diameter of 0.25 mm. We used a commercial controller (WP SLP Controller; P&A Solutions, Seongdong-gu, Seoul, Korea) to sweep voltages and measure the currents of the Langmuir probe.

As the Langmuir probe principle is well described elsewhere [35,36], we briefly explain it in this section. With sweeping voltage, the Langmuir probe measures the voltage–current curve. The plasma potential is derived as the voltage at the peak in the first derivative of the voltage–current curve. We also estimated electron density ($n_{e}$) as

(14) $$n_{e}=\int_{0}^{\infty }f_{e}\left(E\right)dE,$$

and electron temperature ($T_{e}$) as

(15) $$T_{e}=\frac{1}{n_{e}}\int_{0}^{\infty }f_{e}\left(E\right)EdE,$$

respectively, where E is the electron energy and $f_{e}$ is the electron energy distribution function measured by the Langmuir probe.

### 3.2. Results and Discussion

**Figure 5:**
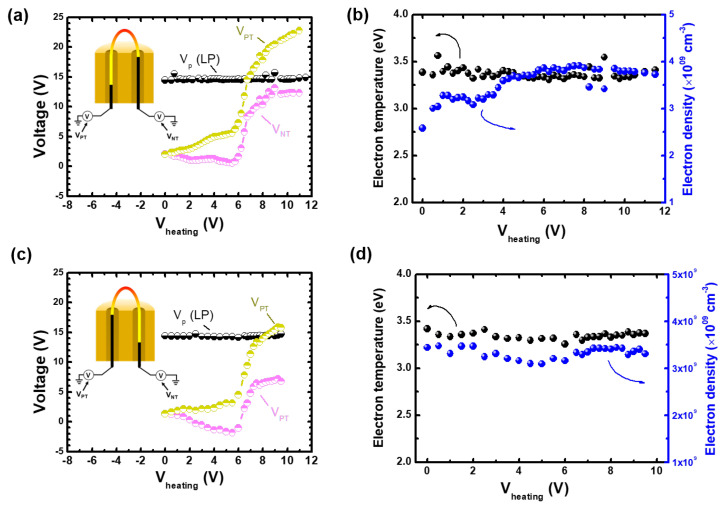
(**a**,**c**) Measured plasma potential (V${}_{p}$(LP)) obtained with Langmuir probe, positive and negative potentials (V${}_{\mathrm{PT}}$ and V${}_{\mathrm{NT}}$) obtained with the emissive probe, and (**b**,**d**) measured electron density and temperature obtained with the Langmuir probe with different emissive probe symmetry: (**a**,**b**) left-sided and (**c**,**d**) right-sided symmetry.

Figure 5a shows the measurement results of plasma potential, V${}_{p}$, using the Langmuir probe and of terminal voltages, V${}_{\mathrm{PT}}$ and V${}_{\mathrm{NT}}$, in the left-sided filament configuration. We summarized the measurement results in Table 1. Here, V${}_{p}$ remained from 14.7 V to 14.8 V with the increase in $V_{\mathrm{heating}}$. The voltage characteristics of the emissive probe in the experiment reproduced the circuit model results shown in Figure 3d well; both terminal voltages exhibited the same behavior with the increase in V${}_{\mathrm{heating}}$. Emission began at V${}_{\mathrm{heating}}$ greater than 6 V. As $V_{\mathrm{NT}}$ was saturated to 12.3 V, as shown in Table 1, and V${}_{\mathrm{PT}}$ increased with the increase in $V_{\mathrm{heating}}$. This corresponded to the circuit model results.

It is noted that the measured V${}_{\mathrm{NT}}$ is close to $V_{p}$(LP) as shown in Figure 5a. However, there is a slight difference between them. Based on the circuit model analysis, this could have resulted from the voltage drop in the small part of the filament inside the ceramic tube, since the voltage drop of a copper wire is negligible. Indeed, the filament of the negative terminal side was inserted into the ceramic tube for the length of a few millimeters. Furthermore, the space charge effect could have affected the difference, as mentioned in Section 1, but it was negligible in this low-plasma-density region [32]; it was effectively larger than the electron density of 10${}^{11}$ cm${}^{-3}$, which was much larger than that in this ICP source, as shown in Figure 5c.

In the case of the right-sided filament configuration, V${}_{\mathrm{NT}}$ became lower than V${}_{p}$, as shown in Figure 5c. The filament insertion of the negative terminal side into the ceramic tube caused the decrease in V${}_{\mathrm{NT}}$. This is the same as the result of the circuit model shown in Figure 3. Hence, the experimental results indicate that the lowest voltage along the plasma-exposed filament region is close to the plasma potential, which corresponds to the circuit model result.

It is noted that electron emission in this experiment did not change plasma. When V${}_{\mathrm{heating}}$ increased, the Langmuir probe measurements revealed that the plasma potential, electron temperature, and electron density were rarely perturbed, as shown in Figure 5b,d.

## 4. Conclusions

This study investigated the voltages along the probe surface and proved which voltage approaches the plasma potential. Using a circuit model, we found that the voltage on the negative terminal side of an emissive probe approaches the plasma potential. To validate this result, we compared the voltages at the positive and negative terminals of an emissive probe and measured the plasma potential with a Langmuir probe in an inductively coupled plasma system. The experimental results reproduced the circuit model results well. Hence, we can conclude that the lowest potential on the plasma-exposed filament provides a close approximation of the plasma potential.

## Figures and Tables

**Table 1:** Summary of measurement results of Langmuir probe (LP), and positive and negative terminals of emissive probe.

	Left-Sided Symmetry	Right-Sided Symmetry
V${}_{p}$ (Langmuir probe)	14.8 V	14.7 V
V${}_{\mathrm{PT}}$ (positive terminal)	22.8 V	6.79 V
V${}_{\mathrm{NT}}$ (negative terminal)	12.3 V	15.8 V

## Data Availability

The data presented in this study are available upon request from the corresponding author.
